# Role of Thermal Process on Self-Assembled Structures of 4′-([2,2′:6′,2″-Terpyridin]-4′-Yl)-[1,1′-Biphenyl]-4-Carboxylic Acid on Au(III)

**DOI:** 10.3390/ijms14035686

**Published:** 2013-03-11

**Authors:** Xiaoqing Liu, Yongli Wang, Xin Song, Feng Chen, Hongping Ouyang, Xueao Zhang, Yingxiang Cai, Xiaoming Liu, Li Wang

**Affiliations:** 1 Department of Physics, Nanchang University, Nanchang 330031, China; E-Mails: liuxiaoqing@ncu.edu.cn (X.L.); sxpoyc@sina.com (X.S.); fengchen10.10@163.com (F.C.); kaede_0120@126.com (H.O.); yxcai@yahoo.com (Y.C.); 2 Department of Chemistry, Nanchang University, Nanchang 330031, China; E-Mail: pikki561@163.com; 3 College of Science, National University of Defense Technology, Changsha 410073, China; E-Mail: xazhang@nudt.edu.cn; 4 Chemical Sciences and Engineering, College of Biological, Jiaxing University, Jiaxing 314001, China; 5 Nanoscience and Nanotechnology Laboratory, Institute for Advanced Study, Nanchang University, Nanchang 330031, China

**Keywords:** dynamic processes, self-assembled structure, hydrogen bond, dipole

## Abstract

The role of dynamic processes on self-assembled structures of 4′-([2,2′:6′, 2″-terpyridin]-4′-yl)-[1,1′-biphenyl]-4-carboxylic acid (l) molecules on Au(III) has been studied by scanning tunneling microscopy. The as-deposited monolayer is closed-packed and periodic in a short-range due to dipole forces. A thermal annealing process at 110 degrees drives such disordered monolayer into ordered chain-like structures, determined by the combination of the dipole forces and hydrogen bonding. Further annealing at 130 degrees turns the whole monolayer into a bowknot-like structure in which hydrogen bonding plays the dominant role in the formation of assembled structures. Such dependence of an assembled structure on the process demonstrates that an assembled structure can be regulated and controlled not only by the molecular structure but also by the thermal process to form the assembled structure.

## 1. Introduction

Self-assembly is a type of process in which a disordered system of pre-existing components forms an organized structure or pattern as a consequence of specific, local interactions among the components themselves, without external direction [1]. Self-assembled monolayers (SAMs) of organic molecules are readily tuned by designed molecular building blocks and can be stabilized by hydrogen bonding, dipole stacking, Van der Waals interactions, π–π stacking or metal coordination between these building blocks [2–14]. To date, SAMs have introduced unprecedented flexibility to tailor interfaces and generate patterned surfaces and have been applied in biology, electrochemistry and electronics, nanoelectromechanical systems and microelectromechanical systems [15–18].

**Figure 1:**
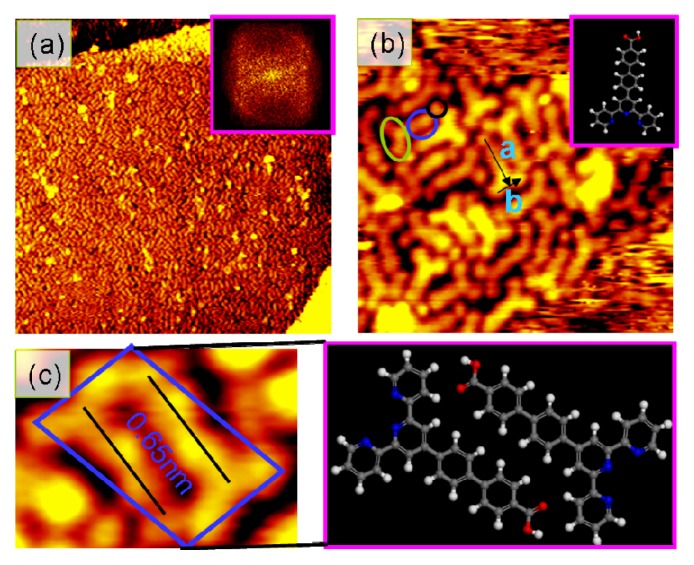
Scanning tunneling microscopy (STM) images of monolayer of l molecules on Au(III) surface. *V*_s_ = −2.6 *V*, *I*_t_ = −30 pA. (**a**) 50 × 50 nm^2^; (**b**) 10 × 10 nm^2^; (**c**) molecular arrangement model building block.

Hydrogen bond, a highly selective, directional and moderately strong intermolecular force [19,20], is widely regarded as an important driving force for the formation of supramolecular self-assemblies and has been successfully used to direct the assembly of molecules into controlled size and shape, such as clusters, chains, triangle, honeycomb and porous networks [2,3,5,7,9,10,13,14,18,21,22]. In an actual system, hydrogen bonds often cooperatively act with the other intermolecular interactions, such as dipole and Van der Waals interactions. Consequently, the minimization of the overall free energy for an assembled system is caused by all the existing intermolecular interactions. Therefore, whether the formation of hydrogen bonds between molecules could be related to the process to form the assembled structure or not when hydrogen bonding and the other intermolecular interactions coexist in a given system. Conjugated 4′-([2,2′:6′,2″-terpyridin]-4′-yl)-[1,1′-biphenyl]-4-carboxylic acid (l) molecule synthesized by ourselves consists of three pyridine rings, two benzenes and one carboxyl [the inset of Figure 1a]. We speculate that dipole force, hydrogen bonds and other intermolecular interactions between l molecules may exist in a given system because of the specific structure of the l molecule. The coexistence of various types of intermolecular interactions in a system provides a feasible testbed to understand the competition between the dipole force, Van der Waals interaction, and H bonding in the formation of assembled structures. The thermal annealing process is a feasible way to break the current balance of intermolecular interaction and let the system reach a new balance so that the organic molecule could form a new assembled structure.

In this paper, the role of thermal processes on self-assembled structures of conjugated 4′-([2,2′:6′, 2″-terpyridin]-4′-yl)-[1,1′-biphenyl]-4-carboxylic acid (l) molecules on Au(III) has been studied by scanning tunneling microscopy (STM). The as-deposited monolayer is closed-packed but lack of long-range periodicity and the building blocks in this stage are dimers of l molecules that are caused by dipole forces. A thermal annealing process at 110 degrees gradually drives such disordered monolayer into ordered chain-like structures in which the building blocks are 4 l molecules and determined by the combination of dipole force and hydrogen bonding. A further annealing process at 130 degree turns the whole monolayer into structures in which hydrogen bonding plays a dominant role in the formation of assembled structure. Such dependence of an assembled structure on the process results from competition of intermolecular interactions among the molecules within the monolayer, which demonstrates that an assembled structure can be regulated and controlled not only by the molecular structure but also by the thermal process to form the assembled structure.

## 2. Results and Discussion

Figure 1 shows the typical STM images for l molecules deposited on Au(III) surface at room temperature. Although the whole surface is covered by l molecules, the herringbone reconstruction in Figure 1a is still observed underneath such molecular layer, suggesting that only one monolayer of l molecules is present on Au surface and there are not any chemical interactions between the molecules and the surface [23]. STM observations also clearly reveal that this monolayer of l molecules on the surface is closed-packed but lack long-range periodicity while the qualities of the STM images are disturbed by the presence of mobilized molecules (bright spots in these images) on the top of the monolayer. The spots in the Fourier transform (FT) image of this STM image [the inset of Figure 1a] indicate that the distributions of the molecules within this monolayer actually are periodic in a short-range. Close examinations demonstrate that each of the molecules on the surface appears as Y-shape features with dimensions of a = 1.45 nm and b = 0.72 nm, which is in good agreement with values (1.46 nm and 0.70 nm) derived from chemical structure of l molecule in the inset, as shown in Figure 1b. Thus, the parts of the Y-shape features highlighted as green circles and blue circles represent the pyridine ring and biphenyl chain of the molecule, respectively. On the other hand, the appearance of the molecules as Y-shape features confirms that the l molecules actually take a lying-down adsorption geometry on the Au surface. Figure 1c also reveals that one l molecule is inclined to couple with the upside-down l molecule to form a dimer with a spacing of 0.65 nm. DFT calculations indicate that the charge of the l molecule distributes unevenly along the spin axis, as a consequence, an electric dipole moment of 1.747 D is formed along the direction from the pyridine ring pointing to the carboxyl group. Moreover, a suspended protrusion within a Y-shaped feature highlighted as black circle can be clearly observed and is assigned to the carboxyl group within a l molecule, which strongly indicates that the hydrogen bondings are absent among these molecules. Therefore, the dipole interactions between the molecules are mainly the driving forces to form the dimers within the monolayer and also account for the presence of a short-range periodicity.

**Figure 2:**
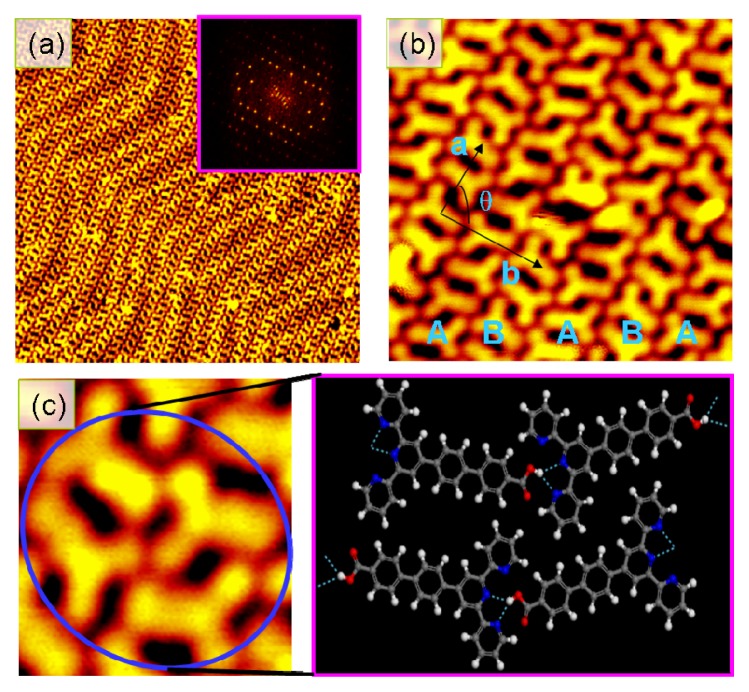
STM images of chain-like monolayer of l molecules on Au(III) surface. (**a**) *V*_s_ = 3.5 *V*, *I*_t_ = 30 pA. 45 × 45 nm^2^; (**b**) *V*_s_ = 2.5 *V*, *I*_t_ = 40 pA. 10 × 10 nm^2^; (**c**) a model for building block. The dash lines denote the O–H: N hydrogen bonds between the l molecules.

Figure 2a shows an STM image for a monolayer of l molecules on Au(III) surface after annealing at 110 degrees for 10 min. The facts that the underneath herringbone reconstruction of Au surface become much clearer and the sharp spots periodically distribute on the FFT image unambiguously reveal that this monolayer of l molecules is well ordered with a long-range periodicity. As shown in Figure 2b, this large-area ordered assembled monolayer consists of two types of molecular chains labeled as A and B. In each type of molecular chains, the l molecules appear as Y-shape features but the direction of any l molecule is opposite to those of two neighboring molecules. The vectors of a basic unit cell for such ordered self-assembled monolayer are described a = 2.05 nm and b = 3.21 nm and the angle between the vectors is 90°. On the other hand, the density for such ordered monolayer, 1.367 molecules/nm^2^, is less than that for the as-deposited disordered monolayer (about 1.530 molecules/nm^2^). This indicates that a portion of the l molecules were desorbed from the surface and at the same time the residual l molecules rearranged themselves into the ordered structures during the annealing process. The building blocks for such ordered monolayer consist of two dimers in two neighboring molecular chains, as shown in Figure 2c. Although two l molecules in a dimer tend to align themselves in parallel, it is obvious that there is an angle between the axis of one molecule and that of the other molecule. Moreover, the separation between the molecules in a dimer is about 1.02 nm and larger than the value (0.65 nm) for a dimer within the disordered monolayer. Such differences in the geometry between a dimer for this ordered monolayer and that for the as-deposited disordered monolayer suggest that dipole interactions between the l molecules cannot solely account for the formation of a dimer within this ordered self-assembled monolayer. Meanwhile, the carboxyl groups within the l molecules are no longer distinguished. The hydrogen bonds are formed between the molecules via the carboxyl groups. It is these hydrogen bonds that connect the dimers on the neighboring molecular chains together and form a long-range periodicity. Thus, the driving forces to form such ordered chain-like self-assembled monolayer is the combination of the dipole forces between the neighboring molecules in the same chains and the O–H: N hydrogen bonds between the neighboring molecules in the neighboring molecular chains.

**Figure 3:**
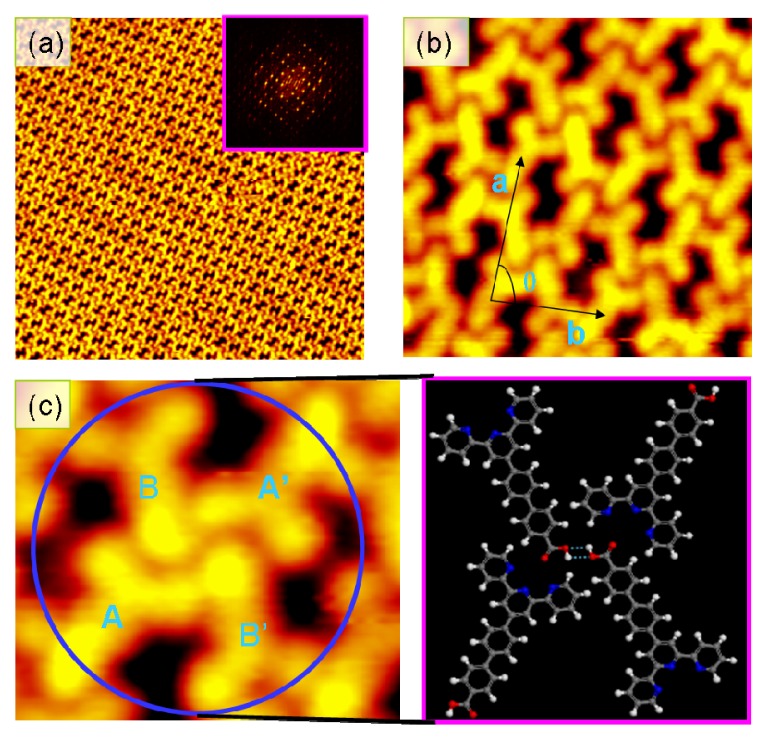
STM images of chain-like monolayer of l molecules on Au(III) surface. (**a**) *V*_s_ = 3.3 *V*, *I*_t_ = 30 pA. 45 × 45 nm^2^; (**b**) *V*_s_ = 2.5 *V*, *I*_t_ = 40 pA. 10 × 10 nm^2^; (**c**) building block and DFT molecular arrangement model.

Further annealing at 130 degrees for 20 min, dramatically changes the structure of a monolayer of l molecules on Au surface. Although the monolayer of l molecules in Figure 3a is still an ordered structure with a long-range periodicity after the annealing process, the basic vectors of a unit cell for this monolayer are derived from the STM images to be a = 2.70 nm, b = 3.21 nm and the angle = 88°. A high-resolution STM image in Figure 3b demonstrates that the building block for this kind of monolayer is a bowknot-like structure consisting of four l molecules but these four molecules do not form dimers as observed in the chain-like assembled structure of l molecules. It is reasonable to deduce that the driving force for the formation of such a bowknot structure does not originate from dipole interaction. In this bowknot-like structure, the pyridine parts of two opposite A and A′ molecules and the carboxyl groups of the other two B and B′ molecules point to the center of the building block together. The proposed model for this bowknot structure clearly reveals that hydrogen bonds highlighted as the cyan dash lines are present between the carbon groups of B and B′, as shown in Figure 3c. Therefore, the driving force to form such a bowknot structure is mainly due to the C–O: H hydrogen bonds between the two l molecules. It is worth noting that the density of the bowknot-like assembled monolayer is 1.038 molecules/nm^2^ and less than that for chain-like assembled monolayer, indicating that a portion of the molecules desorbed from the monolayer during the second annealing process and more spaces are created for the rearrangements of the molecules on the surface to form the bowknot-like structure.

Three self-assembly structures are determined by the competition among the various interactions between l molecules on Au(III) surface. The intermolecular interactions have a distinct hierarchy in their ranges and strengths and are anisotropic due to the spherical asymmetry of l molecules, which therefore allows for flexible engineering of the supermolecular structure while keeping the molecule largely unaffected. The as-deposited structure is mainly caused by the dipole and van der Waals interactions between the molecules. The hydrogen bond between craboxyl and pyridine ring between the neighboring molecules is of greater strength and is located on two ends of l molecules. Therefore, those l dimers tend to align in a certain direction to favor the formation of this kind of hydrogen bond; as a consequence, a long-range ordered chain-like self-assembled structure is formed after a thermal annealing process at 110 degrees. A hydrogen bond between two carboxyl groups is of the order of ten times as large as a van der Waals interaction and is also stronger than the hydrogen bond between craboxyl and pyridine rings. Further thermal annealing at 130 degrees allowed the molecules rearrange themselves to reach the formation of these much stronger hydrogen bonds via the carboxyl groups between l molecules. At the same time, the charge of the l molecules distributes more unevenly along the axis of the bonds after the formation of hydrogen bonds between the carboxyl groups so that the Coulomb interactions are present between the countered the pyridine rings and carboxyl groups. The l molecules are driven by these two kinds of intermolecular interactions to form the bowknot-like structure.

## 3. Experimental Section

The experiments were carried out in a multichamber ultrahigh vacuum (UHV) system housing a SPECS variable temperature STM with a base pressure of less than 2 × 10^−10^ mbar. A gold single crystal (111) (Mateck GmbH, Juelich, Germany) was cleaned by repeated cycles of Ar^+^ bombardment and annealing (~800 K). After several hours of degassing, conjugated 4′-([2,2′:6′,2″-terpyridin]-4′-yl)-[1,1′-biphenyl]-4-carboxylic acid (l) which was prepared via a three-step synthesis using 2-oxo-2-(pyridine-2-yl)ehan-1-ylium and methyl 4′-formyl-[1,1′-biphenyl]-4-carbonxylate as precursors [24], was evaporated from a Knudsen cell at 443 K onto the clean Au surface at room temperature for 1 min. The samples were treated gradually by thermal annealing. The annealing steps were at 363 K, 383 K, 403 K and 423 K for 20 min respectively. STM images were acquired at room temperature with a chemically etched W tip. Positive voltage indicates that the samples were biased positively with respect to the tip. Density functional theory (DFT) calculations were performed using Gaussian 09 with the exact exchange (HF).

## 4. Conclusions

The self-assembled structures of l molecules on Au(III) fabricated by various thermal processes has been studied by scanning tunneling microscopy. The as-deposited monolayer of l molecules is closed-packed and periodic in a short-range. Thermal annealing processes gradually drive such disordered monolayer into chain-like or bowknot-like structures when different annealing temperatures are applied. The dependence of an assembled structure on the thermal process results from the competition between intermolecular interactions, *i.e.*, dipole interaction and hydrogen bonding, among the molecules within the monolayer. Our observations clearly demonstrate that an assembled structure of organic molecules on metal surfaces can be regulated and controlled, not only by molecular structure, but also by the thermal process to form the assembled structure.

## Supplementary Files

Supplementary File 1 Role of Thermal Process on Self-Assembled Structures of 4'-([2,2':6',2''-Terpyridin]-4'-Yl)-[1,1'-Biphenyl]-4-Carboxylic Acid on Au(III) (DOCX, 123 KB)
