# Supplementary material for: Role of Thermal Process on Self-Assembled Structures of 4′-([2,2′:6′,2″-Terpyridin]-4′-Yl)-[1,1′-Biphenyl]-4-Carboxylic Acid on Au(III)
Source: Int J Mol Sci. 2013 Mar 11;14(3):5686–93. doi: 10.3390/ijms14035686 (PMC3634450; doi:10.3390/ijms14035686)
Supplement: Supplementary File 1 — Role of Thermal Process on Self-Assembled Structures of 4'-([2,2':6',2''-Terpyridin]-4'-Yl)-[1,1'-Biphenyl]-4-Carboxylic Acid on Au(III) (DOCX, 123 KB) [file ijms-14-05686-s001.docx]

Supporting Information

Syntheses of l Molecule

**4'-([2,2':6',2''-Terpyridine]-4'-yl)-[1,1'-biphenyl]-4-carboxylic:** 30% NH_3_ solution (1 mL) and NaOH (0.054 g, 1.26 mmol) dissolved in a minimum amount of water were added slowly to a solution of methyl 4'-formyl-[1,1'-biphenyl]-4-carbonxylate (0.15 g, 0.63 mmol) and 2-acetylpyridine (0.14 mL, 1.26 mmol) in ethanol (25 mL). After the addition of the NaOH, the solution turned yellow and after about 1 h lucid brownish red. The solution was stirred vigorously at room temperature in a flask open to air for 24 h, after which water
(50 mL) was added to the solution, a slightly yellow precipitate was obtained. HCl was added to the solution, which was then neutralized alkali to pH = 5. The precipitate was collected by filtration and washed with water. For further purification it was refluxed for 1 h in 25 mL EtOH, and the solid collected by filtration and dried in vacuum. (0.045 g, 20%).

**Scheme S1.** l Molecule synthesis.


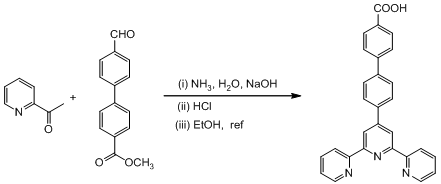


**Figure S1.** Characterization of l Molecule*.* Interrelated maps of l molecule as follows:
^1^H NMR of l molecule (400 MHz, DMSO); ^1^H NMR (400 MHz, DMSO): δ 13.03 (s, 1H, COOH), 8.80 (s, 4H, H^6A^, H^3B^), 8.71 (d, *J* = 6.9 Hz, 2H, H^3A^), 8.17–8.03 (m, 6H, H^3D^, H^2D^, H^3C^), 8.03–7.96 (m, 2H, H^2C^), 7.96–7.87 (m, 2H, H^4A^), 7.56 (d, *J* = 2.2 Hz, 2H, H^5A^).

**Figure S2.** Thermo gravimetric curve of l molecule.


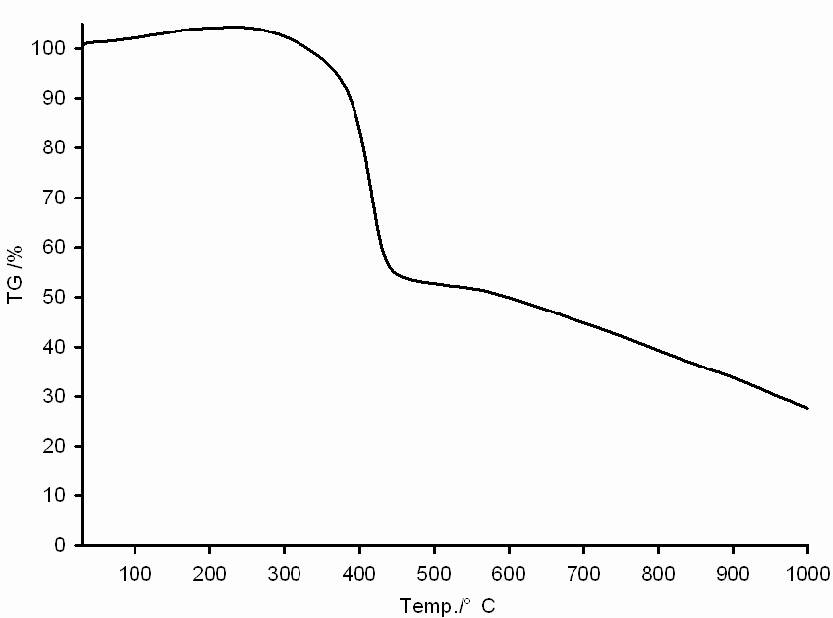


**Figure S3.** Infrared spectroscopy of l molecule.


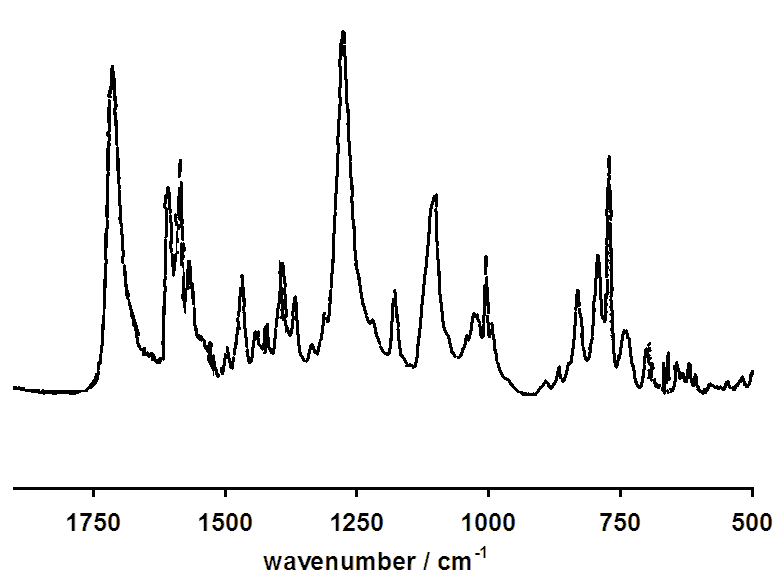


© 2013 by the authors; licensee MDPI, Basel, Switzerland. This article is an open access article distributed under the terms and conditions of the Creative Commons Attribution license (http://creativecommons.org/licenses/by/3.0/).
